# Reelin Is Involved in Transforming Growth Factor-β1-Induced Cell Migration in Esophageal Carcinoma Cells

**DOI:** 10.1371/journal.pone.0031802

**Published:** 2012-02-29

**Authors:** Yi Yuan, Hongyan Chen, Gang Ma, Xiaofeng Cao, Zhihua Liu

**Affiliations:** 1 State Key Laboratory of Molecular Oncology, Cancer Institute, Chinese Academy of Medical Sciences and Peking Union Medical College, Beijing, China; 2 National Key Laboratory of Plant Genomics and Center for Plant Gene Research, Institute of Genetics and Developmental Biology, Chinese Academy of Sciences, Beijing, China; 3 Graduate University of the Chinese Academy of Sciences, Beijing, China; Sun Yat-sen University Cancer Center, China

## Abstract

Reelin (RELN), which is a glycoprotein secreted by Cajal-Retzius cells of the developing cerebral cortex, plays an important role in neuronal migration, but its role in cell migration and cancer metastasis is largely unclear. Here, we showed that cell motility was significantly increased in KYSE-510 cells by TGF-β1 treatment. Moreover, TGF-β1 decreased RELN mRNA expression and overexpression of Reelin at least partly reversed TGF-β1-induced cell migration in KYSE-30 cells. Furthermore, this negative regulation of Reelin expression by TGF-β1 was through Snail, one transcription factor which was induced by TGF-β1 in KYSE-510 cells. RELN promoter activity was reduced in parallel with the induction of Snail after TGF-β1 treatment and Snail suppressed both RELN promoter activity and expression through binding to E-box sequences in the RELN promoter region in ESCC cells. Knockdown of RELN induced cell migration in KYSE-510 cells, together with the increase of mesenchymal markers expression. Taken together, Reelin is an essential negative regulator in the TGF-β1-induced cell migration process, and is suppressed by TGF-β pathway at the transcriptional level through Snail regulation. Therefore, the correlation of Reelin and TGF-β pathway was critical in cancer metastasis, and Reelin could be one potential anti-metastasis target in future clinical practice.

## Introduction

Reelin was known as a large glycoprotein secreted from Cajal-Retzius cells of developing cerebral cortex, and acts as a critical regulator of neuronal migration and layer formation during brain development [1], [2]. Reelin binds to Apolipoprotein E Receptor 2 (ApoER2) and very low-density lipoprotein receptor (VLDLR) [3], and thereby induces phosphorylation of an intracellular adaptor protein, Disabled-1 (Dab1) [4]. The physiological function of Reelin was intensively studied in brain, however, recently, RELN was found to be epigenetically silenced in different cancers including pancreatic [5], gastric [6] and breast cancer [7]. Moreover, the decreased expression of RELN was associated with increased migratory ability, reduced survival and poor prognosis, reduced expression of Reelin is associated with high recurrence rate of hepatocellular carcinoma [8]. In contrast, strong Reelin expression was found to be correlated with high-grade prostate cancer [9].

Esophageal cancer is the sixth leading cause of cancer death worldwide and, interestingly, also the least studied type of tumor [10]. There is an exceedingly high incidence of esophageal squamous cell carcinoma (ESCC) in Asian countries, especially in north and central China [11]. Although 90% of cancer deaths are caused by metastasis [12], the mechanism of cancer metastasis remains poorly defined, and understanding this process will provide great promise for cancer therapy. Epithelial-mesenchymal transition (EMT) is thought to be a crucial step of metastasis [13]. During embryo development, organogenesis and wound repair, EMT is tightly controlled temporally and spatially, but when EMT is dysregulated, it will cause fibrosis and invasion and metastasis of carcinoma [14], [15], [16]. During EMT, the epithelial cells lose the polarity and become more migratory, fibroblast-like cells with concomitant loss of expression of epithelial markers, such as cytokeratins, E-cadherin, and desmoplakin [17]. EMT can be induced by many cytokines, and transforming growth factor-β (TGF-β) was found to be critical for EMT induction. In response to TGF-β1, the Smad-dependent signaling pathway cooperates with other Smad-independent pathways to regulate target genes, including Snail family, ZEB family, Twist, etc [18], [19], [20]. However, the role of TGF-β in esophageal carcinogenesis and its signaling pathway in EMT process are not yet understood.

Although it is known that Reelin expression can be regulated in many important processes, very little is known with respect to how the expression of Reelin is regulated in esophageal epithelial cells and its role in ESCC metastasis and TGF-β signaling. In this study, we demonstrated that Reelin over-expression suppressed TGF-β1-induced motility of KYSE-30 cells, and its expression can be regulated by Snail. Our results provide the first evidence that Reelin was involved in TGF-β signal pathway, which contributes to cancer metastasis and could be useful for anti-cancer strategies.

## Results

### TGF-β1 Induces Cell Morphologic Changes and Migration in Esophageal Carcinoma Cell Lines

**Figure 1:**
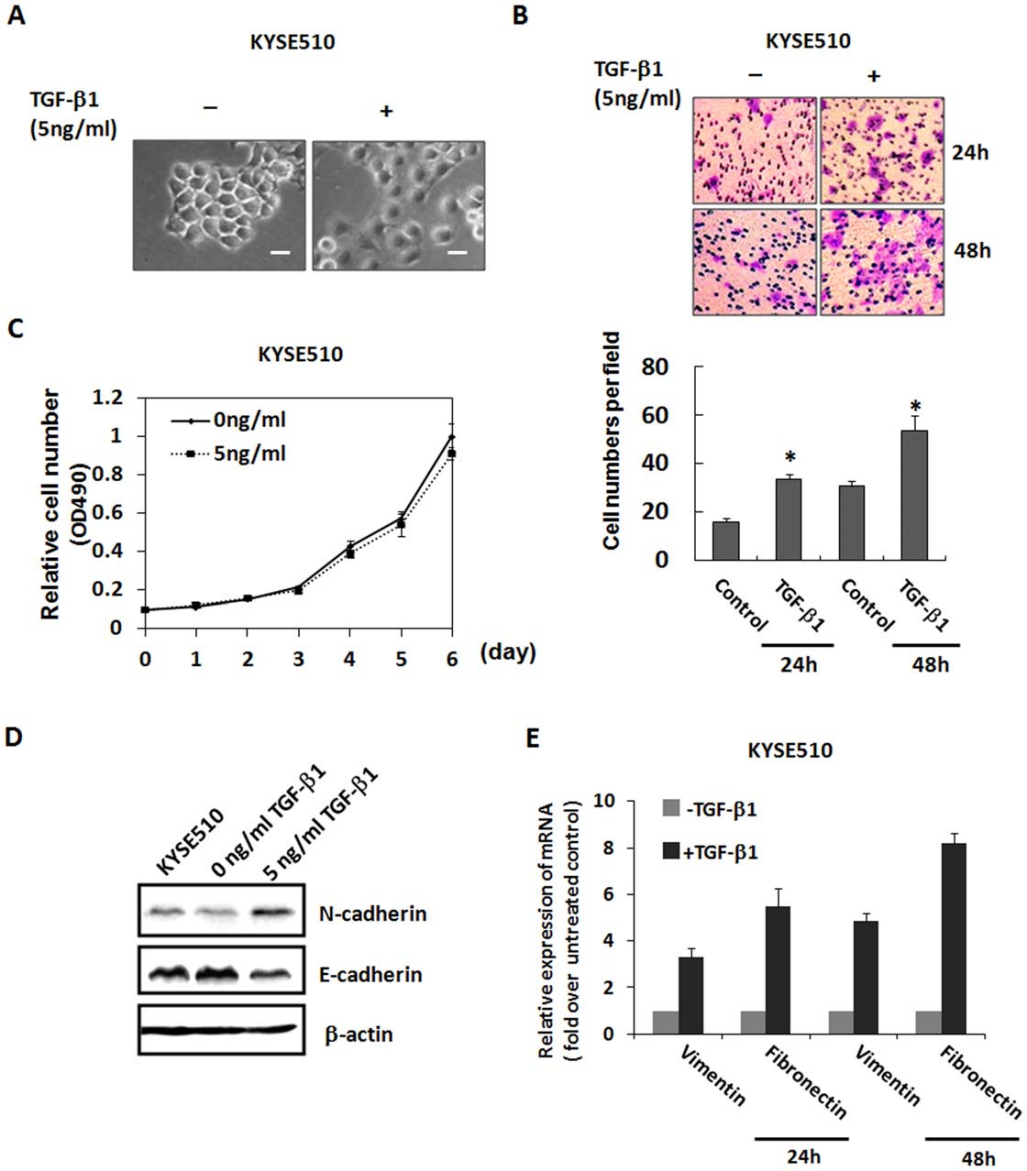
TGF-β1 induced cell morphologic changes and migration in KYSE-510 cells. Cells were treated with 5 ng/ml TGF-β1 for indicated time. **A**: After treatment of TGF-β1 or control, morphologic phenotypes and KYSE-510 cells were examined under an inverted phase-contrast microscope. *Scale bars*, 20 µm. **B**: Transwell assay showing TGF-β1-induced cell migration in KYSE-510 cells. Cells were stained with crystal violet (*top*). The bar graph shows the relative number of migrated cells from three independent experiments (mean±SE, *bottom*). *, *p*<0.05. **C**: Measurement of cell viability and proliferation KYSE-510 after TGF-β1 treatment by MTT assay. **D**: Western blot for N-cadherin and E-cadherin using whole cell extract at 48 hours after TGF-β1 treatment in KYSE510 cells. β-actin served as the loading control. **E**: RT-qPCR analysis showing the mRNA expression levels of some EMT markers in KYSE-510 cells with or without TGF-β1 treatment for indicated time. Data represent the mean ± SD of triplicate experiments.

Since the role of TGF-β in esophageal carcinogenesis is not yet understood, we investigated whether TGF-β1 treatment induced ESCC cells to mesenchymal transition and further elucidated the underlying mechanism responsible for the process. KYSE-30 and KYSE-510 cells were treated with TGF-β1 and morphologic phenotypes were examined under an inverted phase-contrast microscope. As shown in Figure S1A and Figure 1A, TGF-β significantly induced KYSE-30 and KYSE-510 cell spreading after 24 hours treatment. Transwell assays proved the motility of KYSE-510 cells was increased (Figure 1B), excluding the possibility of cell number increase (Figure 1C). TGF-β1 suppressed E-cadherin expression and increased N-cadherin expression (Figure 1D). Since the protein level of Vimentin and Fibronectin was low in KYSE-510 cells, we could not detect the expression by Western blot; therefore the mRNA expression level was determined in this study. As shown in Figure 1E, TGF-β1 significantly increased the mRNA expression levels of mesenchymal markers: Vimentin and Fibronectin at 24 and 48 hours after treatment. Although TGF-β1 significantly suppressed KYSE-30 cells growth (Figure S1B), the mRNA expression of Vimentin was also increased (Figure S1C).

### TGF-β1 Suppressed RELN Expression and TGF-β1-Induced Migration Was Suppressed by Reelin Overexpression

**Figure 2:**
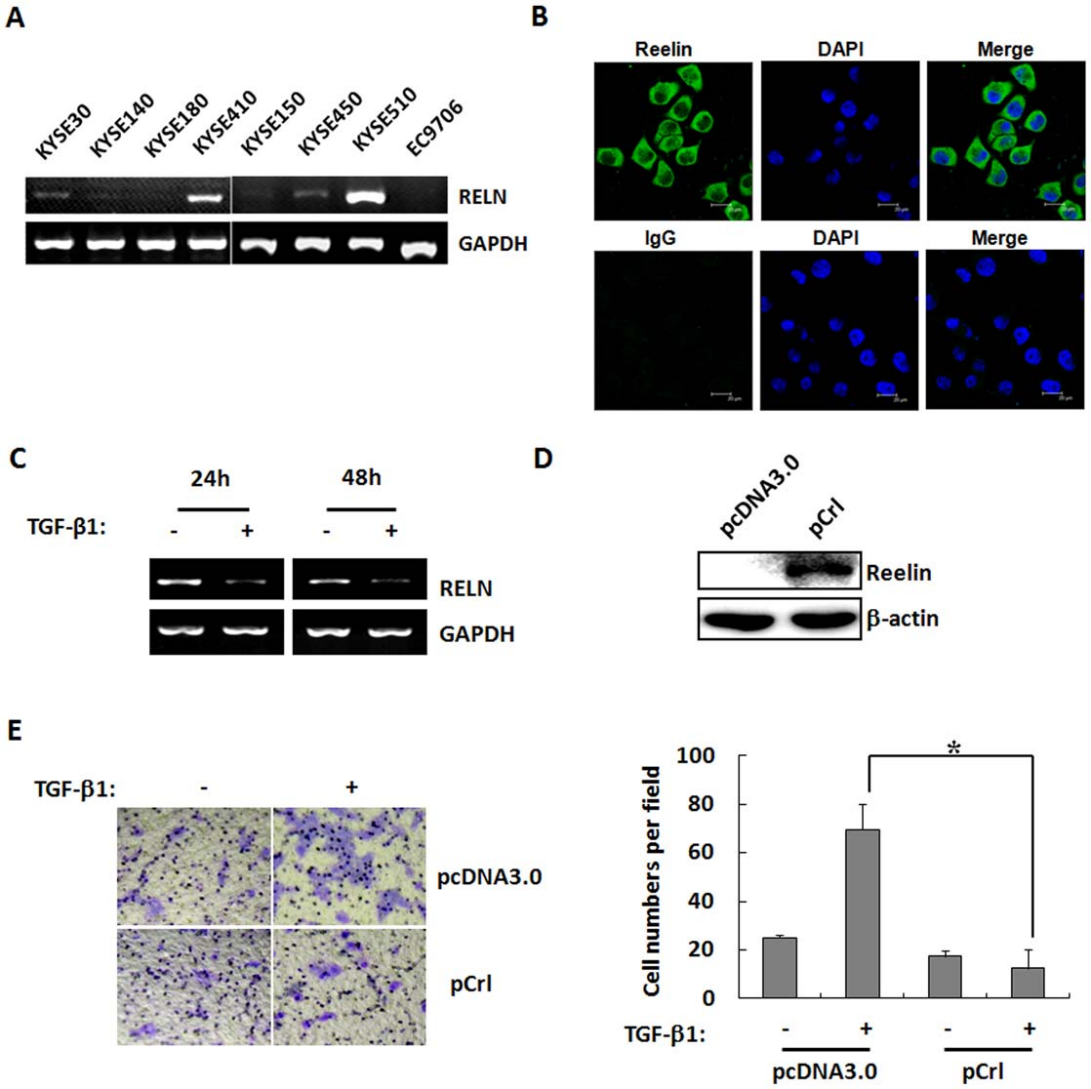
TGF-β1 suppressed RELN expression and transient transfection of Reelin blocked TGF-β1-induced cell migration. **A**: RT-PCR analysis showing RELN mRNA expression in eight ESCC cell lines, GAPDH was used as an internal control. **B**: Reelin protein was localized in cytoplasm in KYSE-510 cells by immunostaining. Mouse IgG was used as isotype negative control. *Scale bar*: 20 µm. **C**: KYSE-510 cells were treated with 5 ng/ml TGF-β1, RELN mRNA expression was examined by RT-PCR, and GAPDH was used as an internal control. **D**: Western blot for Reelin protein using whole cell extract at 48 hours after reelin (pCrl) transfection in KYSE-30 cells. β-actin served as the loading control. **E**: 5 ng/ml TGF-β1 was added in media for 24 hours after pCrl transfection and Transwell assay was performed at 48 hours after transfection (*left*). The bar graph shows the relative number of migrated cells from three independent experiments (*right*). Data represent the mean ± SD of triplicate experiments. *, *p*<0.05.

Reelin was reported to be important in neural development and cancer metastasis, thus we speculated that Reelin might be involved in TGF-β1-induced cell migration. We first detected the mRNA expression of RELN in ESCC cell lines. As shown in Figure 2A, RELN mRNA expression was low in most of ESCC cell lines except for KYSE-410 and KYSE-510 cells. Furthermore, immunofluorescence showed that Reelin was mostly localized in cytoplasm in KYSE-510 cells (Figure 2B). Importantly, we found that RELN mRNA level was significantly decreased after 5 ng/ml TGF-β1 treatment in KYSE30 and KYSE510 cells (Figure S1D and Figure 2C). In contrast, RELN mRNA expression is relatively low in KYSE-30 cells, so we overexpressed reelin in this cell line to test whether Reelin plays a functional role in TGF-β1-induced cell migration, we transiently transfected reelin expression vector (pCrl) followed by TGF-β1 treatment in KYSE-30 cells (Figure 2D), and found that Reelin overexpression significantly abolished TGF-β1-induced cell migration, as shown by Transwell assay (Figure 2E). These results suggested that Reelin is critical in blocking the TGF-β1-induced cell migration.

### TGF-β1 Transcriptional Regulated RELN Expression in KYSE-510 Cells

**Figure 3:**
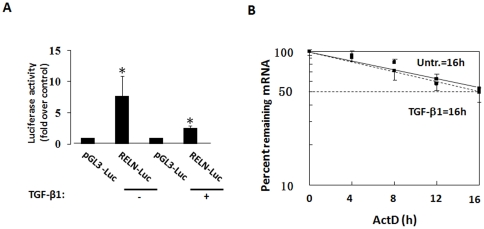
TGF-β1 treatment decreased RELN transcriptional activity, but did not affect RELN mRNA stability. **A**: TGF-β1 down-regulated the RELN promoter activity. KYSE-510 cells were transiently transfected with pGL3-basic and the RELN promoter-luciferase construct, and simultaneously treated by 5 ng/ml TGF-β1 at 24 hours after transfection. Data represent the mean ± SD of triplicate experiments. *, *p*<0.05. B. KYSE-510 cells were treated with or without 5 ng/ml TGF-β1 for 1 hour followed by actinomycin D (*Act D*; 5 µg/ml) treatment for the indicated time. RELN mRNA level was measured by RT-qPCR (*left*) and plotted on a logarithmic scale to calculate the time required for each mRNA to reach one-half of its initial abundance (*right*). Dotted line represents RELN mRNA expression after TGF-β1 treatment and straight line represents RELN mRNA expression of untreated control.

To investigate whether TGF-β1 suppressed RELN expression at transcriptional level, we constructed a luciferase reporter gene vector pGL3-RELN carrying a core promoter of human RELN extending from −514 bp to +76 bp based on a previous report [21] and transfected into KYSE-30 and KYSE-510 cells followed by TGF-β1 treatment for 48 hours, and found that the luciferase activity of RELN promoter was reduced comparing with control (Figure 3A). To further evaluate whether TGF-β1 has the effect on RELN mRNA stability, KYSE-510 cells were treated with TGF-β1 for 1 hour prior to the addition of actinomycin D (ActD), and quantitative RT-PCR analysis was performed. RELN mRNA stability with or without TGF-β1 treatment was not different and the half-life of RELN mRNA is ∼16 hours (Figure 3B).

### Transcriptional Regulation of RELN by Snail

**Figure 4:**
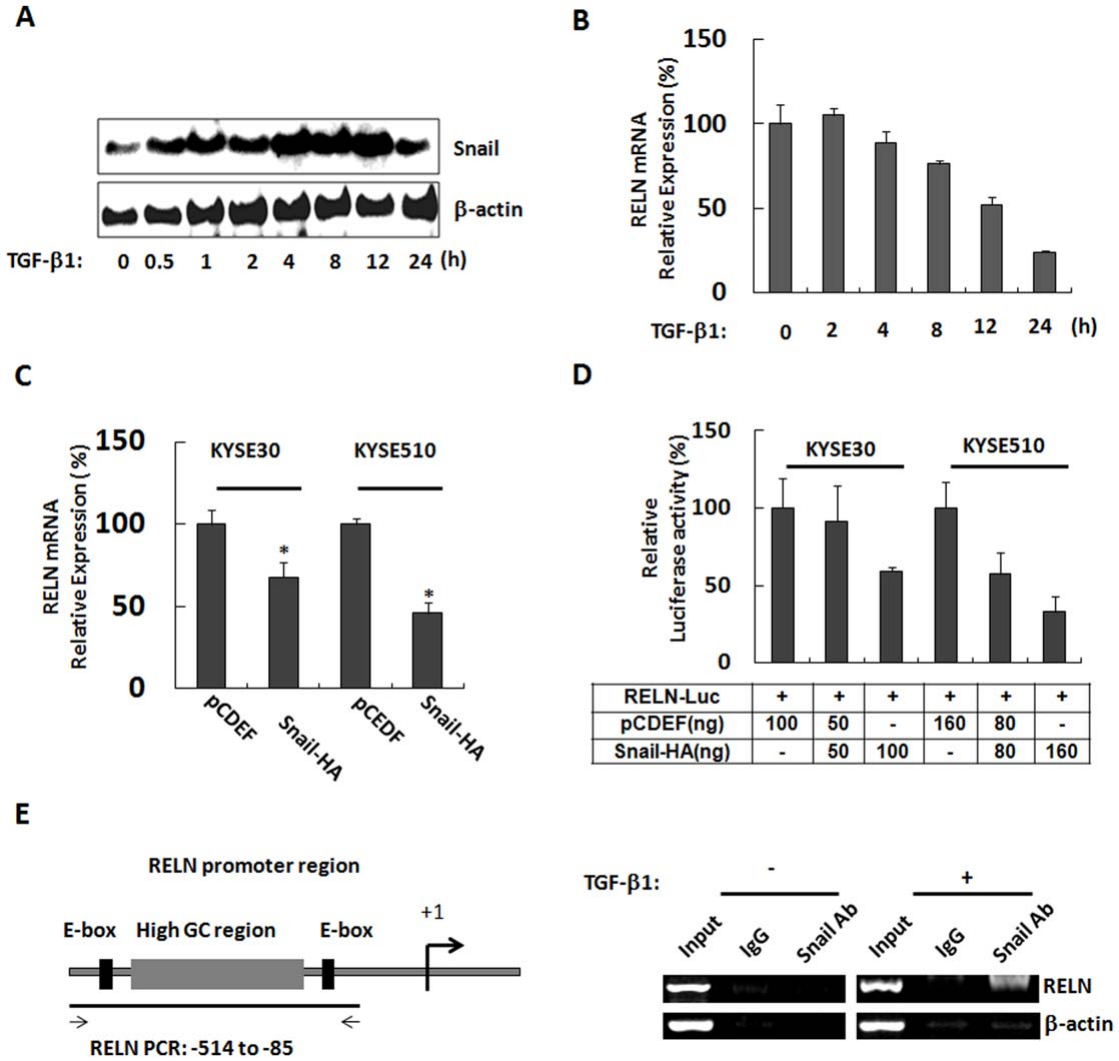
Snail regulates RELN expression in ESCC cells. **A&B**: Snail protein was increased (**A**) and RELN mRNA was decreased (**B**) in a time-dependent manner after 5 ng/ml TGF-β1 treatment in KYSE-510 cells. β-actin served as the loading control in Western blot. **C**: RT-qPCR analysis showing that Snail down-regulates RELN mRNA expression in KYSE30 and KYSE-510 cells. Data represent the mean ± SD of triplicate experiments. *, *p*<0.05. **D**: Snail down-regulates RELN promoter activity in KYSE-30 and KYSE510 cells in a dose-dependent manner. Data represent the mean ± SD of triplicate experiments. **E**: Snail binds to the RELN promoter after TGF-β1 treatment in KYSE-510 cells. Schematic representation of RELN promoter region, +1 = transcription start site (*left*). ChIP assay was carried out by using a rabbit anti-Snail antibody or the rabbit IgG as described in *Materials and Methods*. The presence of sequences corresponding to RELN promoters and β-actin were analyzed, and β-actin served as the negative control (*right*).

It is well known that Snail plays important roles in TGF-β1 induced EMT process, and its expression was increased after TGF-β1 treatment in many cancer cell lines [22], [23], [24]. After TGF-β1 treatment, Snail expression was increased (Figure 4A) and RELN expression was decreased (Figure 4B) in a time-dependent manner in KYSE-510 cells. To assess regulation of Snail on RELN expression, Snail overexpression construct pcDEF-Snail was transfected into KYSE-30 and KYSE-510 cells, mRNA of RELN was significantly decreased in KYSE-30 and KYSE-510 cells (Figure 4C). Furthermore, co-transfection of pcDEF-Snail and the RELN promoter region (−514/+76) led to a dose-dependent decrease in luciferase activity (Figure 4D), indicating that Snail has a repressive effect on RELN promoter.

Because Snail binds to specific sequences that contain a central core sequence of 5′-CACCTG-3′ in the promoter of E-cadherin gene, which was termed as E-box [25], [26] and subsequently represses the expression of E-cadherin, thus we speculated that there might be some Snail binding sites in the RELN promoter region. Fortunately, 2 specific sequences have been identified in RELN promoter region which exactly matched E-box sequence, located from −466 to −460 bp (5′-CAGCTG-3′) and −206 to −201 bp (5′-CACGTG-3′) relative to the RNA start site (Figure 4E, *left*). We subsequently performed a ChIP assay to test this hypothesis, and found that Snail binds to the region extending from −514 to −85 bp of RELN promoter very weakly; however, very interestingly, Snail could strongly bind to the same sequences of RELN promoter region after TGF-β1 treatment (Figure 4E, *right*).

### RELN knockdown endowed KYSE-510 cells with stronger motility

**Figure 5:**
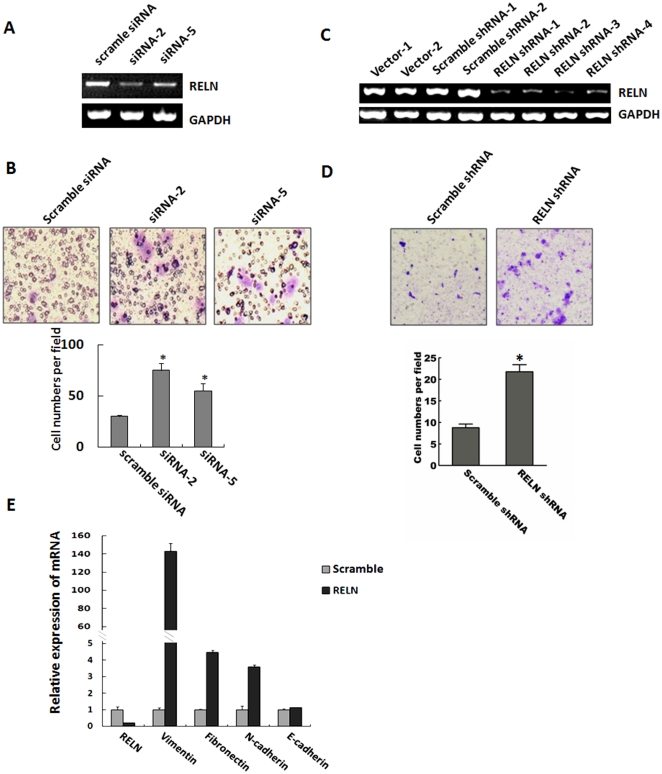
Role of Reelin in cell migration. **A**: RT-PCR analysis showing RELN mRNA level in RELN and scramble siRNAs transfected cells, and GAPDH was used as an internal control. **B**: Transwell assay showing that RELN knockdown increased cell migration. Cells were stained with crystal violet (*left*). The bar graph shows the relative number of migrated cells from three independent experiments (mean+SE, *right*). *, p<0.05. **C**: RT-PCR analysis showing RELN mRNA level in RELN shRNA and scramble clones. **D**: Transwell assay showing cell migration of the RELN shRNA and scramble clones. Cells were stained with crystal violet (*top*). The bar graph shows the relative number of migrated cells from three independent experiments (mean+SE, *bottom*). *, *p*<0.05. **E**: RT-qPCR showing the mRNA expression of some EMT markers in RELN shRNA and scramble clones. Data represent the mean ± SD of triplicate experiments.

To characterize the physiological function of RELN, we performed transient knockdown experiments using 2 pairs of siRNAs (siRNA-2 and 5) targeting RELN in KYSE-510 cells. The results demonstrated that RELN knockdown induced a marked increase in cell migration (Figure 5A&B). We then constructed specific RELN shRNA vectors and obtained RELN shRNA stable clones (Figure 5C). Consistent with the transient transfection, stable knockdown of RELN also increased cell motility (Figure 5D). Meanwhile, the mRNA levels of some mesenchymal markers Vimentin, Fibronectin and N-cadherin dramatically increased in the RELN shRNA stable clone, whereas the epithelial marker E-cadherin remained unchanged (Figure 5E). These data clearly demonstrated that RELN functions as a cell migration suppressor.

### RELN Expression in Esophageal Carcinoma Tissues

**Figure 6:**
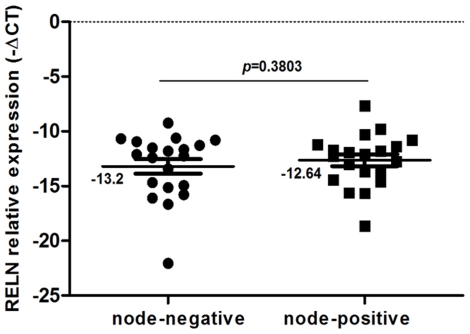
RT-qPCR analysis showing the relative expression of RELN mRNA in 40 human ESCC tissues. The term −ΔCt was used to describe the relative expression level of RELN (−ΔCt = CtGAPDH-CtRELN). No significant difference was detected in the mean value of RELN expression between these two groups (*p* = 0.3803).

We subsequently detected RELN mRNA expression in 40 pairs of human ESCC tissues by RT-qPCR (Figure 6), and the term −ΔCt was used to describe the expression level of RELN. We explored the relationship between the expression level of RELN in ESCC tissues and various clinicopathological parameters (Table 1). The median expression of RELN was −13.5 in the 21 cases with advanced stage (stage III and IV) disease, whereas the median expression was −12.2 in the 19 cases with early-stage (stage I and II) disease. However, no significant difference was observed between the 20 cases of ESCC with lymph node metastasis, the median expression of RELN was −12.6, and the other 20 non-metastasis ESCC cases, the median expression was −13.2. (*p* = 0.3803, Table 2).

**Table 1:** The clinicopathologic characteristics of 40 ESCC patients.

Clinicopathologic characteristics	Case numbers
Age (median)	38–72 (57)
Sex (male/female)	30/10
Differentiation (well/moderate/poor)	14/17/9
Stage (I/II/III/IV)	1/18/20/1
Tumor depth (submucosa/muscularis propria/adventitia)	4/9/27
Lymph node metastasis (negative/positive)	20/20

**Table 2:** Relationship between RELN mRNA expression level and clinicopathological features in 40 primary ESCC samples.

Features	Casenumbers	RELN expression(−ΔCt)	*P*-value
Stage(TNM)			
Stage I/II	19	−12.2	0.146
Stage III/IV	21	−13.5	
Lymph node metastasis			
negative	20	−13.2	0.3803
positive	20	−12.6	

RELN expression means mRNA expression in human ESCC tissues described in Figure 6.

Student's *t* test was used to examine the difference of RELN mRNA levels.

## Discussion

Reelin was necessary for migration of neuron from their site of origin to their final destination and was expressed in embryonic and adult mammalian tissues [27], [28]. Migration underlies many physiological and pathological processes including embryonic development, wound repair, and tumor metastasis [29], [30]. In this study, we demonstrated that Reelin probably functions as a cell migration-related gene. We first found that Reelin was involved in TGF-β1-stimulated migration. Downregulation of Reelin expression was detected in the KYSE-510 cells after TGF-β1 treatment. Moreover, knockdown of RELN in the same cell line led to dramatic increase in the expressions of several mesenchymal markers including vimentin, fibronectin and N-cadherin, suggesting that loss of RELN could endow cells with some mesenchymal traits and stronger mobility (Figure 5). Considering the constant expression of E-cadherin in those cells, the knockdown of RELN in KYSE-510 cells was likely to go through a partial EMT transition, which is proposed to assist migration and invasion [31]. Epithelial cells can migrate during development not only by complete EMT, but also through collective migration where epithelial cells physically and functionally connected as a group [32], [33], [34]. One of the similarities between EMT and collective migration was the acquisition of an invasive and motile phenotype; however E-cadherin expression was decreased in EMT and remained unchanged in collective migration. In this study, knockdown of RELN expression induced ESCC cell migration maintaining E-cadherin expression which functions in cell-cell adhesion, but the mechanism of cell-cell adhesion in the process of ESCC cell migration is still not clear and needs to be further investigated.

Notably, our findings reveal an unknown link between Snail, which triggers EMT process and favors tumor progression [35], [36], and RELN gene. Snail is a zinc finger transcriptional repressor which has a highly conserved C-terminal domain, containing from four to six C2H2 type zinc fingers and bind to the E-box [37]. Snail expression was low in KYSE-510 cells, but was dramatically increased after TGF-β1 treatment (Figure 4A), and RELN mRNA expression was decreased in a time-dependent manner (Figure 4B). Thereby, an inverse correlation between RELN mRNA and Snail protein levels is observed upon TGF-β1 treatment. Interestingly, there are 2 E-box sequences in RELN promoter region, and we identified the recruitment of transcriptional repressor Snail to the RELN promoter after TGF-β1 treatment. And the E-box located from −466 to −460 bp (5′-CAGCTG-3′) was also identified as a cyclic AMP responsive element binding protein (CREB) binding site which has not been functionally characterized at this point [38]. The mechanism study showed that Snail elevated responding to the TGF-β1 treatment, then anchored to the E-box of RELN promoter, and finally obstructed the expression of RELN. In addition, we observed elevated expression of RELN mRNA after treatment with the histone deacetylase inhibitor, TSA, in ESCC cells (data not shown), and Snail was also reported to be interacted with HDAC1 to repress E-cadherin expression [39], [40]. And RELN promoter was epigenetically regulated through DNA methylation [21]. Equally important in this context is the interplay between methylation, chromatin structure and histone deacetylation. Thus, we speculated that Snail might interact with various classes of histone deacetylases (HDACs) in complexes that repress RELN transcription and perhaps induce histone deacetylation in ESCC cells.

Recently, several groups reported that loss of functional Reelin was implicated in motility and invasion of pancreatic, gastric, and breast cancer [5], [6], [7]. Our previous study showed that Reelin was dysregulated in ESCC samples [41]. In this study, knockdown of Reelin expression considerably enhanced mobility of ESCC cells, which is consistent with previous reports [5]. Unfortunately, further examination on ESCC specimens failed to find a significant correlation between RELN expression and clinical metastasis status. One possible explanation is that there are limited amount of metastatic cancer cells in the tested specimens. Another reason rests on multiple steps of metastasis. Because metastasis consists of a serial of procedures and we have not determined in which steps RELN may be involved. Therefore, the result in ESCC tissues is not necessarily in conflict with the finding that RELN suppressed cell migration of human ESCC cells.

In summary, Reelin is involved in TGF-β1-mediated cell migration in ESCC cells, and the TGF-β1-induced migration could be suppressed by Reelin expression. We further demonstrated that Snail can regulate Reelin expression through binding to Reelin promoter region *in vivo* after TGF-β1 treatment and decreased RELN promoter activity in a dose-dependent manner. And we showed that knockdown of Reelin induced the expression of mesenchymal markers and increased cell migration in KYSE510 cells. Our results provide the first evidence linking Reelin to TGF-β signal pathway, which contribute to cancer metastasis, and it is helpful for anti-cancer strategies.

## Materials and Methods

### Ethics Statement and Tissue Specimens

This study was approved by the ethical committees of the Chinese Academy of Medical Sciences Cancer Institute and the First Affiliated Hospital of Anhui Medical University, and informed consent was obtained from each patient. Forty primary human ESCC tissue specimens were provided by the First Affiliated Hospital of Anhui Medical University (Anhui Province, China). All the tissues were obtained at the time of surgery and immediately stored in liquid nitrogen until use. None of the patients had received radiotherapy or chemotherapy before surgery. Patients diagnosed with metastasis had lymph node metastasis verified by pathological analysis. For all the samples, clinicopathologic characteristics (age, gender, differentiation, stage, tumor depth, and lymph node metastasis) are shown in Table 1 [42].

### Cell Lines-Growth and Treatment

Human ESCC cell lines KYSE-30, KYSE-140, KYSE-150, KYSE-180, KYSE-410, KYSE-450, KYSE-510, and EC9706 were all established from human ESCC patients. Among them, the KYSE series were generous gifts from Dr. Y. Shimada at Hyogo College of Medicine (Hyogo, Japan) and EC9706 was established and maintained in our laboratory [43], [44], [45]. Cells were maintained in RPMI-1640 supplemented with 10% fetal bovine serum and antibiotics. Subconfluent cells were treated with 5 ng/ml TGF-β1 (R&D system, Radnor, USA).

### RNA Interference and Transfection

Small interfering RNAs (siRNA; Qiagen, *Mississauga*, *Canada*) targeting the RELN (Hs_RELN_2_HP siRNA, AGGAACGCTTTGACAGTGAA; Hs_RELN_5_HP siRNA, ATGGGCGGCGTCAGCTAATTA) and the control small interfering RNA scramble siRNA (AATTCTCCGAACGTGTCACGT) were used at 25 nmol/L. Stable silencing of RELN was achieved using the short hairpin RNA-based vector pGCsi-U6/neo/GFP (Genechem, Shanghai, China) with the target sequence (AAGGAACGCTTTGACAGT-GAA or ATGGGCGGCGTCAGCTAATTA) and the negative control sequence (TTCTCCGAACGTGTCACGT). Hiperfect (Qiagen) was used for siRNA transfection according to the manufacture's protocols.

### Plasmid Construction and Transfection

The Snail-HA expression vector which expresses Snail under control of a pcDEF backbone was cloned in our lab, and the reelin expression vector (pCrl; generous gift from Dr. Tom Curran, The Children's Hospital of Philadelphia, Philadelphia, PA, USA) which expresses full-length reelin under control of a cytomegalovirus promoter in a pcDNA3 backbone (neomycin-resistant) was first cloned by D'Arcangelo et al [46]. Lipofectamine 2000 (Invitrogen, Carlsbad, USA) was used for plasmid transfection.

### Production of Stable Cell Lines

KYSE-510 cells were plated on 60-mm dishes, cultured for 24 hours at 37°C, then transfected with 3 µg of negative shRNA or RELN shRNA expression vector. Cells were reseeded on three 100-mm dishes at 24 hours after transfection, and cultured in 10% fetal bovine serum of RPMI-1640 with 400 µg/ml G418. After a 2-week selection, the cell colonies were picked into 24-well plates for stable cell lines setup, cultured in 10% fetal bovine serum of RPMI-1640 with 200 µg/ml G418, and then screened with RT-PCR.

### Transwell Assay

For migration assay, KYSE-30 or KYSE-510 cells trypsinized and seeded in medium without serum; then 1×10^5^ cells plated into the upper chambers of cell culture inserts (24-well type, 8-µm pore size, Corning, NY, USA), which were placed in medium containing 20% fetal bovine serum served as chemoattractant. After 24 or 30 hours of incubation, cells attached to the upper side of the filter were mechanically removed, and cells that had migrated to the undersurface of the membrane were fixed and stained with crystal violet. Digital images were obtained from the membranes, and three random fields were counted. The results were averaged in three independent experiments. Transwell assays were also performed after 5 ng/ml TGF-β1 treatment.

### Immuofluorescence Analysis

Cells on chamber slides were fixed with 4% paraformaldehyde for 30 min at room temperature and permeablized with 0.2% Triton X-100 for 2 min. The cells were then treated sequentially with Reelin antibody (Santa Cruz) in PBS at 4°C overnight followed 3 washes in PBS and subsequent incubation with appropriate fluorescent secondary antibody conjugates for 30 minutes. Images were collected using Leica Confocal Laser Scanning microscopy (Leica Microsystems, Wetzlar, Germany).

### RNA Isolation and PCR Analysis

Total RNA was isolated by using Trizol reagent (Invitrogen) according to the manufacturer's instructions. Conventional reverse transcription-PCR (RT-PCR) and quantitative RT-PCR (RT-qPCR) were carried out using PrimeScript One-Step RT-PCR kit and One-Step SYBR PrimeScript RT-PCR kit (TaKaRa, Dalian, China) respectively. The primers used include: RELN, forward 5′-TGCTGCAATACAGCGTCAACAA-3′ and reverse 5′-CGACCTCCACATGGTCCAAAG-3′; Vimentin, forward 5′-GAGAAC-TTTGCCGTTGAAGC-3′ and reverse 5′-GCTTCCTGTAGGTGGCAATC-3′; Fibronectin, forward 5′-CAGTGGGAGACCTCGAGAAG-3′ and reverse 5′- TCCCTC-GGAACATCAGAAAC-3′; N-cadherin, forward 5′- ACAGTGGCCACCTACAAAG-G-3′ and reverse 5′-CCGAGATGGGGTTGATAATG-3′; E-cadherin, forward 5′-TGCCCAGAAAATGAAAAAGG -3′ and reverse 5′- GTGTATGTGGCAATGCGT-TC-3′; GAPDH, forward 5′-ACCACAGTCCATGCCATCAC-3′ and reverse 5′-TCCACCACCCTGTTGCTGTA-3′. Actinomycin D (ActD; Sigma, St. Louis, USA) was used to inhibit RNA synthesis in KYSE-510 cells. The expression of each gene relative to GAPDH was calculated using a modified method of delta Ct (2^−ΔCt^) described by Lehmann and Kreipe [47].

### Chromatin Immunoprecipitation

Chromatin immunoprecipitation analysis (ChIP) was performed with Chromatin Immunoprecipitation Assay Kit (Millipore, Billerica, USA) using rabbit anti-Snail (Santa Cruz biotechnology, Santa Cruz, USA) or control rabbit IgG (Zhongshanjinqiao, Beijing, China) according to the manufacturer's instructions. The primers used include: RELN, forward 5′-AAAAACAGGGCACACTGACG -3′ and reverse 5′-TCCACACCT-TCTTAAAGCCCC-3′; β-actin, forward 5′-GTTCTGCTCCGGGAATCACAG-3′ and reverse 5′- ACGCGCTGCAAAGAGCCCCGC-3′.

### Luciferase Reporter Gene Assay

The minimal promoter region of RELN [21] was constructed. Cells were plated on 96-well plates, grown to a density of 30∼50% and transfected with the RELN promoter construct or empty vector (pGL3-Basic) using Lipofectamine 2000. Forty-eight hours after transfection, luciferase activity was measured in triplicate using the Dual- Luciferase Assay System (Promega, Madison, USA) and normalized for *Renilla* luciferase activity in corresponding samples. Luciferase reporter gene assays were also performed in the presence of 5 ng/ml TGF-β1.

### Western Blot

Cell lysates were size-fractionated by SDS-PAGE and transferred onto polyvinylidene difluoride membrane. Protein bands were detected by chemiluminescence using the ECL system (Vigorous Biotech, Beijing, China) according to the manufacture's protocol. Mouse anti-N-cadherin (R&D), rabbit anti-E-cadherin (Santa Cruz), rabbit anti-Snail (Santa Cruz), mouse anti-Reelin (G10) (Millipore), and mouse anti-β-actin (Sigma) were used.

### Statistics

Some data are shown as means ± SD from the number of independent experiments as indicated. Statistical analysis was performed using Student's *t* test. All statistical analyses were performed using SPSS software. Differences were considered significant at *P*<0.05.

## Supporting Information

Figure S1 **TGF-β1 induced cell morphologic changes and suppressed RELN expression in KYSE-30 cells.** Cells were treated with 5 ng/ml TGF-β1 for indicated time. A: After treatment of TGF-β1 or control, morphologic phenotypes and KYSE-30 cells were examined under an inverted phase-contrast microscope. Scale bars, 20 µm. B: MTT assay showing the cell viability and proliferation of KYSE-30 after TGF-β1 treatment. C: RT-qPCR analysis showing the mRNA expression levels of Vimentin in KYSE-30 cells with or without TGF-β1 treatment for indicated time. Data represent the mean ± SD of triplicate experiments. D: RELN mRNA expression was examined by RT-qPCR, and GAPDH was used as an internal control.
(TIF)
