# Supplementary figures and images for: Reelin Is Involved in Transforming Growth Factor-β1-Induced Cell Migration in Esophageal Carcinoma Cells
Source: PLoS One. 2012 Feb 29;7(2):e31802. doi: 10.1371/journal.pone.0031802 (PMC3290530; doi:10.1371/journal.pone.0031802)

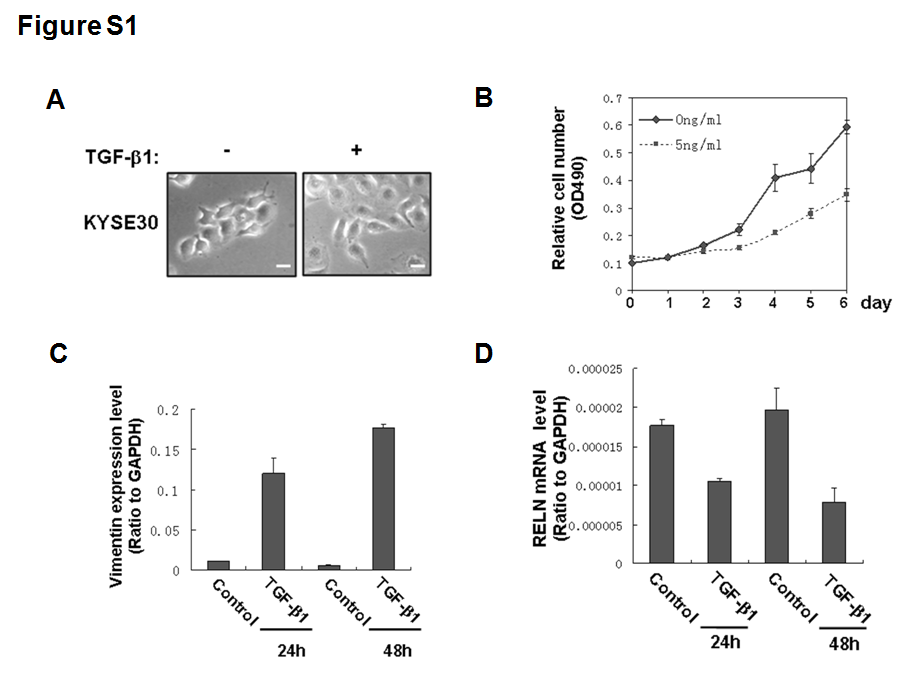

Supplement: Figure S1 — TGF-β1 induced cell morphologic changes and suppressed RELN expression in KYSE-30 cells. Cells were treated with 5 ng/ml TGF-β1 for indicated time. A: After treatment of TGF-β1 or control, morphologic phenotypes and KYSE-30 cells were examined under an inverted phase-contrast microscope. Scale bars, 20 µm. B: MTT assay showing the cell viability and proliferation of KYSE-30 after TGF-β1 treatment. C: RT-qPCR analysis showing the mRNA expression levels of Vimentin in KYSE-30 cells with or without TGF-β1 treatment for indicated time. Data represent the mean ± SD of triplicate experiments. D: RELN mRNA expression was examined by RT-qPCR, and GAPDH was used as an internal control. (TIF) [file pone.0031802.s001.tif]
